# The Mine Locomotive Wireless Network Strategy Based on Successive Interference Cancellation

**DOI:** 10.3390/s151128257

**Published:** 2015-11-09

**Authors:** Liaoyuan Wu, Jianghong Han, Xing Wei, Lei Shi, Xu Ding

**Affiliations:** School of Computer and Information, Hefei University of Technology, Hefei 230009, China; E-Mails: wliaoyuan@hfut.edu.cn (L.W.); hanjh@hfut.edu.cn (J.H.); shilei@hfut.edu.cn (L.S.); dingxu@hfut.edu.cn (X.D.)

**Keywords:** interference management, successive interference calcellation, mine locomotives

## Abstract

We consider a wireless network strategy based on successive interference cancellation (SIC) for mine locomotives. We firstly build the original mathematical model for the strategy which is a non-convex model. Then, we examine this model intensively, and figure out that there are certain regulations embedded in it. Based on these findings, we are able to reformulate the model into a new form and design a simple algorithm which can assign each locomotive with a proper transmitting scheme during the whole schedule procedure. Simulation results show that the outcomes obtained through this algorithm are improved by around 50% compared with those that do not apply the SIC technique.

## 1. Introduction

In the coal mining industry, the mine locomotive plays a very important role in the underground tunnel. When mine locomotives travel along tunnels, we may hope to acquire the real-time information about the underground environment and locomotives’ working data. For example, many researchers focus their attentions on monitoring systems of mine locomotives [1,2]. The goal of using this information to make locomotives work in a more safe and sound situation is, to a great extent, based on the communication quality between locomotives and the control center.

Since the underground tunnels are often in a severe environment, which makes the quality of communication fluctuate all the time, the current monitoring system of mine locomotive is still using the national railway signal concentrated blocking system [3]. However, the locomotives are traveling continuously in tunnels, which means if we want to use the collected data to achieve a better monitoring, we can only use the wireless communication between locomotives and access points (APs) equipped along the tunnels. Moreover, the data packets transferred may have image or video information encapsulated in them, which makes the data volume extremely large. Therefore, designing specific and efficient communication schemes for underground mine locomotives is greatly needed.

The mine locomotive wireless networks are definitely mobile, and the IEEE 802.11 are often applied as the communication protocol. For example, to the vehicular ad hoc networks (VANETs), the commercial communicating protocol is 802.11p [4]. However, the IEEE 802.11 standard is based on carrier-sense multiple access (CSMA) with collision avoidance (CA) [5], which may not be efficient in wireless networks. That is why some researchers focus on wireless mobile network schemes based on time-triggered architecture (TTA) [6]. For example, Sahoo *et al.* proposed a time-slot-based medium access protocol which they named CCC-MAC protocol [7]. The protocol can be used in VANETs, and simulations show it can be used well in different vehicular density scenarios. Yu and Biswas proposed a novel MAC protocol, which can self-configure TDMA protocol, self-organize the MAC layer, and can enhance vehicle safety for vehicle-to-vehicle communication [8].

In underground tunnels with a severe environment, it is more difficult for locomotives communicating with APs. The requirements of achieving a better communication quality and transmitting data fast present challenges to researchers. It is obvious that the network capacity will be improved if we use some powerful wireless communicating techniques, such as OFDM [9,10], CDMA [11,12], and so on. These techniques all share one common property, that is, they try to divide the intact channel into several orthogonal sub channels through which transmissions can be conducted simultaneously. Therefore, the total throughput of the channel can be improved, which has been proved by previous research [13,14,15]. Nevertheless, if several transmissions need to be done in the same sub channel at once, none of them may succeed due to the network congestion. Hence, corresponding solutions to this problem, known as interference avoidance techniques, came into reality. Moreover, in recent years, certain interference cancellation techniques are also well studied. Compared with interference avoidance techniques, interference cancellation techniques allow several transmitters transmit data through the same sub channel at the same time by canceling out the interference rather than scheduling them to complete their tasks according to certain measures. Among all interference cancellation techniques, the successive interference cancellation(SIC) [16,17] is widely used since it is easy to implement while being able to achieve good performance. For example,

Jiang *et al.* proposed an optimal cross-layer algorithm with SIC and showed it can increase the throughput of the whole network by 47% [18], Shi *et al.* proposed an optimal base station placement algorithm based on SIC and showed the algorithm can achieve about 25% improvement [19].

The realization of SIC is not simple but based on some conditions. When a receiver receives a mixed signal from several transmitters, it should be able to decode every transmitter’s signals from the mixed signal, and the signals should be decoded one by one, that is, the receiver firstly tries to decode the strongest signal from the mixed signal and remove it, then tries to decode the second strongest signal from the remaining mixed signal. This process will be repeated until all signals are decoded or the mixed signal can not be decoded any more. Because of the complexity of the process, using SIC in a wireless network will result in a high computational complexity, especially in the mobile wireless network where the transmitting nodes keep moving. Until very recently, seldom work has been done to design SIC-based optimal algorithm for mobile wireless networks.

In this paper, we design the mine locomotive wireless communicating scheme using SIC and which is based on TTA. We first give the problem model in Section 2. From the problem model we can see it is a non-convex problem and can not be solved directly. In Section 3 we introduce our strategy based on SIC. In this section, we also prove some theorems. According to these theorems, we are able to reshape our model into a new form. Simulations in Section 4 shows our strategy can make the network throughput improve by about 50%.

## 2. The Problem Model

**Figure 1:**
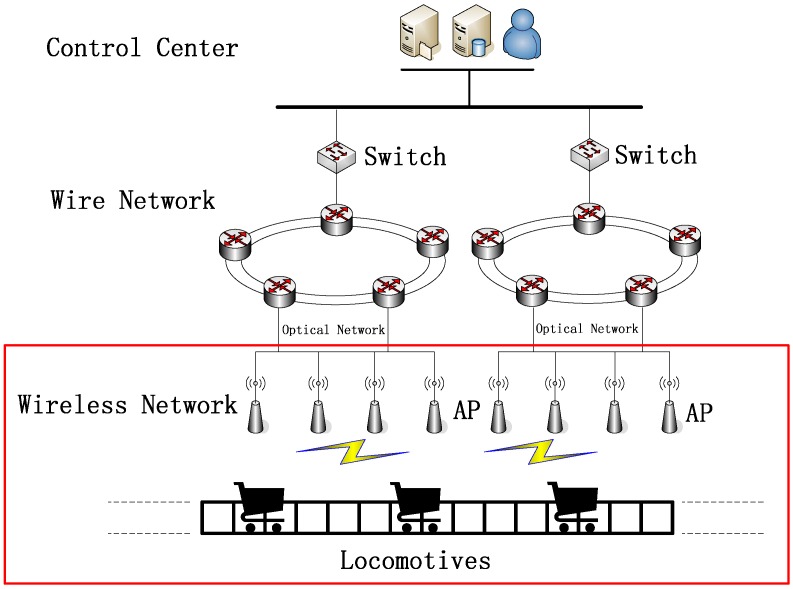
A typical mine locomotive wireless network environment.

A typical mine locomotive wireless network environment is shown in Figure 1. The access points (APs) will be equipped on the side of tunnels. They will collect information from locomotives and send them to the ground monitoring station by wire networks. Locomotives will travel in these tunnels and pass through these APs one by one. When a locomotive pass through a AP, it will transmit its data to the AP directly through wireless network. In this paper, we will only consider how the locomotive transmits its data to one AP. Since under the underground environment the security demand is high, we only consider the one-hop wireless network environment.

**Figure 2:**
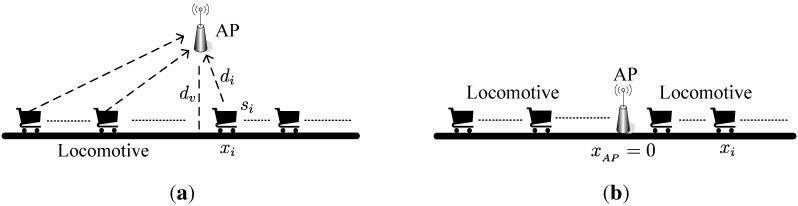
The straight underground tunnel model. (**a**) Original underground tunnel model; (**b**) One-dimensional underground tunnel model.

Consider a mine locomotive wireless network environment with *n* locomotives and one AP in a straight tunnel as shown in Figure 2a. Denote *N* as the set of locomotives. Denote $s_{i}\left(s_{i}\in N\right)$ as the *i*-th locomotive. Denote t(t∈T) as a time point in the whole scheduling time *T*. Denote $d_{i}\left(t\right)$ as the distance between $s_{i}$ and the AP at time *t*. Denote $x_{i}\left(t\right)$ as the coordinate of $s_{i}$ on the tunnel at time *t*. Denote $d_{v}$ as the vertical distance between AP and the tunnel. Denote $R_{T}$ the receiving radius of the AP. $R_{T}$ can also be considered as the maximum transmission power of each locomotive. In the next section, we will give the calculation method for it. Since in the real underground tunnel environment we have $d_{v}\ll R_{T}$, we will suppose $d_{v}\approx 0$ and $x_{i}\left(t\right)=d_{i}\left(t\right)$. That is, we consider one-dimensional area and let the coordinate AP $x_{{}_{AP}}=0$, as shown in Figure 2b. Denote $t_{s_{i}}$ and $t_{s_{i}}^{\prime }$ as the start time and the end time when the $s_{i}$ enters the AP’s range and when it leaves. Then, we have $|x_{i}\left(t_{s_{i}}\right)|=x_{i}\left(t_{s_{i}}^{\prime }\right)=R_{T}$. Denote $v_{i}\left(t\right)\left(t_{s_{i}}\leq t\leq t_{s_{i}}^{\prime }\right)$ as the speed of $s_{i}$ at time *t*. We have Equations (1) and (2).

(1) $$\begin{array}{c} x_{i}\left(k\right)=-R_{T}+\int_{t_{s_{i}}}^{k}v_{i}\left(t\right)dt\;\;\left(s_{i}\in N\right) \end{array}$$

(2) $$\begin{array}{c} 2R_{T}=\int_{t_{i}}^{t_{s_{i}}^{\prime }}v_{i}\left(t\right)dt\;\;\left(s_{i}\in N\right) \end{array}$$

We will use the SIC technique to optimize the whole network. By using SIC, the AP can receive several locomotives’ data simultaneously. However, to use SIC technique correctly, the AP should decode its receiving data sequentially. That is, it should decode these data from the strongest signal to the weakest signal one by one until all signals are decoded or one signal can not be decoded. So even using SIC, the AP may not receive from all locomotives at the same time. Define a one-zero variable

$$\theta_{i}\left(t\right)=\left\{\begin{array}{cc} 1: & \text{if}\;s_{i}\;\text{transmits}\;\text{data}\;\text{to}\;\text{the}\;\text{AP}\;\text{at}\;\text{time}\;t\\ 0: & \text{otherwise} \end{array}\right.$$

Denote $g_{i}\left(t\right)$ as the channel gain from $s_{i}$ to the AP at time *t*. Denote *λ* as the path loss index. We have

(3) $$\begin{array}{c} g_{i}\left(t\right)=\min\{|x_{i}\left(t\right){|}^{-\lambda },1\}\;\;\left(s_{i}\in N,t_{s_{i}}\leq t\leq t_{s_{i}}^{\prime }\right)\; \end{array}$$

Denote $P_{i}\left(t\right)$ as the transmission power of $s_{i}$ at time *t*. Denote $P_{max}$ and $P_{min}$ as the maximum and the minimum power the locomotive can have. We have $P_{min}\leq P_{i}\left(t\right)\leq P_{max}$. Denote $N_{0}$ as the noise ratio. Denote $\sigma_{i}\left(t\right)$ as the signal-to-interference-plus-noise-ratio (SINR) of $s_{i}$ at time *t* under SIC. Then, it can be calculated as

(4) $$\begin{array}{c} \sigma_{i}\left(t\right)=\frac{\theta_{i}\left(t\right)g_{i}\left(t\right)P_{i}\left(t\right)}{N_{0}+\sum_{g_{j}\left(t\right)\leq g_{i}\left(t\right)}\theta_{j}\left(t\right)g_{j}\left(t\right)P_{j}\left(t\right)}\;\;\left(s_{i},s_{j}\in N\right)\; \end{array}$$

Suppose all locomotives have the same minimum data transmission rate requirement and denote it as *r*. For each $s_{i}$, the average data rate over all scheduling time should be no more than $\left(\int_{t_{s_{i}}}^{t_{s_{i}}^{\prime }}\theta_{i}\left(t\right)W\log_{2}\left(1+\sigma_{i}\left(t\right)\right)d\left(t\right)\right)/\left(t_{s_{i}}^{\prime }-t_{s_{i}}\right)$, where *W* is the channel bandwidth. We will maximize scaling factors $K_{i}$ such that each locomotive $s_{i}$ can transmit data to the AP with rate $K_{i}r$. Among the whole scheduling time, the AP can receive data from locomotives as much as possible. Then, we have the following problem.

(5) $$\begin{array}{c} \begin{array}{cc} \max & \sum_{s_{i}\in N}K_{i}r\\ s.t. & \left\{\begin{array}{cc} g_{i}\left(t\right)=\min\{|x_{i}\left(t\right){|}^{-\lambda },1\} & \left(s_{i}\in N,t_{s_{i}}\leq t\leq t_{s_{i}}^{\prime }\right)\\ \sigma_{i}\left(t\right)=\frac{\theta_{i}\left(t\right)g_{i}\left(t\right)P_{i}\left(t\right)}{N_{0}+\sum_{g_{j}\left(t\right)\leq g_{i}\left(t\right)}\theta_{j}\left(t\right)g_{j}\left(t\right)P_{j}\left(t\right)}\geq \beta & \left(s_{i},s_{j}\in N,t\in T\right)\\ K_{i}r_{i}\leq \frac{\int_{t_{s_{i}}}^{t_{s_{i}}^{\prime }}\theta_{i}\left(t\right)W\log_{2}\left(1+\sigma_{i}\left(t\right)\right)d\left(t\right)}{t_{s_{i}}^{\prime }-t_{s_{i}}} & \left(s_{i},s_{j}\in N,t\in T\right)\\ g_{i}\left(t\right),\sigma_{i}\left(t\right),K_{i}\geq 0,\theta_{i}\left(t\right)\in \left\{0,1\right\} & \left(s_{i}\in N,t\in T\right) \end{array}\right. \end{array} \end{array}$$

where $x_{i}\left(t\right)$, $g_{i}\left(t\right)$, $\theta_{i}\left(t\right)$, $\sigma_{i}\left(t\right)$, *t* and $K_{i}$ are all variables. This formulated problem is a non-convex problem, which cannot be solved directly.

## 3. The Optimal Locomotive Communication Strategy Based on SIC

In Section 2, we propose the problem and give the original mathematical model. However, it is a non-convex problem and cannot be solved directly. In this section, we will transform the original model into a model which can be solved directly. We first introduce some theorems.

### 3.1. Some Theorems

**Theorem 1.**  Denote $\alpha_{max}$ as the maximum number of the locomotives that the AP can communicate simultaneously by using SIC, then we have $\alpha_{max}=1+\log_{1+\beta }\frac{P_{max}}{\beta N_{0}}$.

**Proof.**  Suppose at time *t* there are *α* locomotives transmitting their data simultaneously to the AP. Suppose the power of the received signals satisfies $g_{1}\left(t\right)P_{1}\left(t\right)<g_{2}\left(t\right)P_{2}\left(t\right)<\cdots <g_{\alpha }\left(t\right)P_{\alpha }\left(t\right)$, based on the SIC technique, we must have,

$$\begin{array}{c} \begin{array}{cc} g_{1}\left(t\right)P_{1}\left(t\right) & =\beta N_{0},\\ g_{2}\left(t\right)P_{2}\left(t\right) & =\beta \left(N_{0}+g_{1}\left(t\right)P_{1}\left(t\right)\right)=\beta \left(N_{0}+\beta N_{0}\right)=\beta \left(1+\beta \right)N_{0},\\ g_{3}\left(t\right)P_{3}\left(t\right) & =\beta \left(N_{0}+g_{2}\left(t\right)P_{2}\left(t\right)+g_{1}\left(t\right)P_{1}\left(t\right)\right)=\beta \left(N_{0}+\beta N_{0}\left(1+\beta \right)+\beta N_{0}\right)=\beta {\left(1+\beta \right)}^{2}N_{0},\\ \cdots & \\ g_{\alpha }\left(t\right)P_{\alpha }\left(t\right) & =\beta {\left(1+\beta \right)}^{\left(\beta -1\right)}N_{0}. \end{array} \end{array}$$

Then, we have $\alpha =1+\log_{1+\beta }\left(\frac{P_{\alpha }\left(t\right)g_{\alpha }\left(t\right)}{\beta N_{0}}\right)$. Since $P_{\alpha }\left(t\right)\leq P_{max}$ and $g_{\alpha }\left(t\right)=\min\left\{x_{\alpha }{\left(t\right)}^{-\lambda },1\right\}\leq 1$, so we have $\alpha \leq \alpha_{max}=1+\log_{1+\beta }\left(\frac{P_{max}}{\beta N_{0}}\right)$.

From theorem 1, we can see that, based on the SIC technique, the number of the locomotives that the AP can communicate with simultaneously has some relationship with the power that the locomotives use. The higher the power is, the larger the number is. However, from theorem 2, we will see that we can not let the power be arbitrarily large.

**Theorem 2.**  To an AP we have $R_{T}={\left(\frac{\beta {\left(1+\beta \right)}^{\left(\alpha_{max}-1\right)}N_{0}}{P_{max}}\right)}^{-\frac{1}{\lambda }}$.

**Proof.**  Similar to the proof of theorem 1, we have $g_{\alpha_{max}}\left(t\right)P_{\alpha_{max}}\left(t\right)=\beta {\left(1+\beta \right)}^{\left(\alpha_{max}-1\right)}N_{0}$. That is,

(6) $$\begin{array}{c} x_{\alpha_{max}}\left(t\right)={\left(\frac{\beta {\left(1+\beta \right)}^{\left(\alpha_{max}-1\right)}N_{0}}{P_{\alpha_{max}}\left(t\right)}\right)}^{-\frac{1}{\lambda }}\leq {\left(\frac{\beta {\left(1+\beta \right)}^{\left(\alpha_{max}-1\right)}N_{0}}{P_{max}}\right)}^{-\frac{1}{\lambda }}=R_{T}. \end{array}$$

From theorem 2, we can see the receiving radius $R_{T}$ has some relationship with the maximum number of the locomotives transmitting simultaneously. The larger the number is, the smaller $R_{T}$ is. Based on theorems 1 and 2, we should select a suitable $P_{max}$ for the whole network, so that the AP can communicate with enough locomotives simultaneously with a suitable receiving radius.

The following theorems will help us to translate the original continuous problem into a discrete problem. We first introduce theorem 3.

**Theorem 3.**  Suppose in time t a new locomotive $s_{j}$ enters the receiving radius of AP, and can transmit with an old locomotive $s_{i}$ together. Then, during the whole scheduling period, we can always find suitable transmitting powers $P_{i}\left(t\right)$ and $P_{j}\left(t\right)$ so that the two locomotives can transmit together.

**Figure 3:**
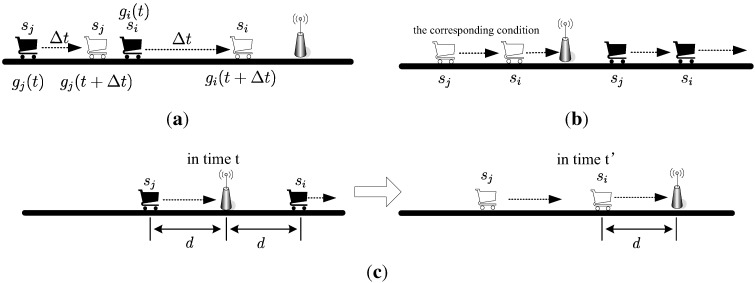
The three conditions for theorem 3. (**a**) The two locomotives are coming closer to the AP; (**b**) The two locomotives are moving away from the AP; (**c**) One locomotive is coming closer to the AP while the other is leaving.

**Proof.**  We consider three different conditions, as shown in Figure 3.

In Figure 3a, the two locomotives are coming closer to the AP. Suppose, in time *t*, we have $\sigma_{j}\left(t\right)=\frac{g_{j}\left(t\right)P_{j}\left(t\right)}{N_{0}+g_{i}\left(t\right)P_{i}\left(t\right)}\geq \beta$ and $\sigma_{i}\left(t\right)=\frac{g_{i}\left(t\right)P_{i}\left(t\right)}{N_{0}}\geq \beta$, where $P_{i}\left(t\right),P_{j}\left(t\right)\leq P_{max}$. In time (t+Δt), since we have $g_{i}\left(t+\Delta t\right)>g_{i}\left(t\right)$ and $g_{j}\left(t+\Delta t\right)>g_{j}\left(t\right)$, we can find suitable $P_{i}\left(t+\Delta t\right)$ and $P_{j}\left(t+\Delta t\right)$, so that $\sigma_{i}\left(t+\Delta t\right),\sigma_{j}\left(t+\Delta t\right)\geq \beta$.

In Figure 3b, the two locomotives are leaving with the AP. Since we have supposed that during the first condition (that is, the two locomotives are coming closer to the AP), we can always find suitable $P_{i}\left(t\right)$ and $P_{j}\left(t\right)$. Then, in the second condition, we can always find an corresponding condition in the first period, so that $s_{i}$ and $s_{j}$ can transmit together.

In Figure 3c, one locomotive is coming closer to the AP while the other is leaving. The most special situation is that ,in time *t*, where the distances between the two locomotives to the AP are equal. To prove that, we can let the two locomotives go back to time $t^{\prime }$. We have proved that in time $t^{\prime }$ the two locomotives can transmit together (the first condition). Since $g_{j}\left(t^{\prime }\right)<g_{j}\left(t\right)$ and $g_{i}\left(t^{\prime }\right)=g_{i}\left(t\right)$, then we can find suitable $P_{i}\left(t\right)$ and $P_{j}\left(t\right)$, so that $\sigma_{i}\left(t\right),\sigma_{j}\left(t\right)\geq \beta$.

In the proof of theorem 3, we consider the situation with two locomotives. However, this theorem can also be applied to the situation of more than two locomotives.

Theorem 3 indicates that we can divide the whole scheduling time *T* into several segments. In each segment, some locomotives will be in the radius of the AP and travel along the tunnel, but no locomotive will enter or leave the range of AP. That is, the number of the locomotives will not change during one segment. Denote *m* as the number of segments for the whole scheduling time *T*. Denote $\tau_{i}\left(i=1\ldots m\right)$ as the *i*-th segment. The length of segment *i* can be expressed as $|\tau_{i}|$, then we have $\sum_{i=1}^{m}\left|\tau_{i}\right|=T$.

Based on the SIC technique, the locomotives can divide $\tau_{i}$ into several time slots, and the locomotives which can transmit together will be arranged into one time slot. Based on theorem 3, during one segment, the arranged time slots do not need to be changed. The only thing we need to change is the sending power of each locomotive. In the start of $\tau_{i}$, new locomotives will enter or old locomotives will leave the radius of the AP. Thus, we only need to arrange time slots in the beginning of each segment.

**Theorem 4.**  If we set suitable power for each locomotive during the whole scheduling time, then the optimal result will be the same no matter the speed is changing or not.

**Proof.**  The optimal result is decided by SINR. The channel gain and the power play important roles in SINR. Different speed will make the locomotive $s_{i}$ in different position in time *t*, and then make the channel gain $g_{i}\left(t\right)$ different. Since in theorem 3 we have proved that we can always find suitable power during the whole scheduling time. Then we can also find suitable power so that no matter the channel gain changes, the SINR is the same, which will prove theorem 4.

Theorem 4 can help us largely to simplify the original problem. In fact, theorem 4 also satisfies the real environment, where all locomotives may have the same speed. In the real environment, though the speed may change a little sometime, we can ignore these changes. Based on these theorems, we can propose our algorithm.

### 3.2. New Model and the Algorithm

To design the algorithm, we suppose that we know all starting times for each locomotive entering the radius of the AP. Since we also suppose that all locomotives have the same speed *v* during the scheduling time, then each locomotive’s position $x_{i}\left(t\right)$ can be calculated. The main idea of our algorithm is based on three basic steps as follows:

- Divide the whole scheduling time into several segments.
- Divide each segment into several time slots, arrange these time slots for locomotives. If a locomotive is in a time slot, it means the locomotive can transmit in this time slot. Otherwise, it means the locomotive can not transmit.
- To each locomotive, give the transmitting power scheme when it transmits.

This algorithm can be implemented on the AP. Each time when some locomotive is entering or leaving the range of the AP, it will try to communicate with it and then the AP will perform the calculation. After that, the AP will tell all locomotives how the transmitting power scheme they will use during the whole scheduling period. If there is no new locomotive entering or leaving, there is no need to do new calculation. The following will discuss these steps in detail.

To the first step, based on theorem 3, we know that if some locomotives can transmit together, then they can always transmit together during the whole period by adjusting their transmitting power so the key time to divide the scheduling time into segments is the time when a locomotive enters the radius of the AP or leaves. That is, we will divide the scheduling time into segments based on each locomotive’s entering time $t_{s_{i}}$ and leaving time $t_{s_{i}}^{\prime }$.

**Figure 4:**
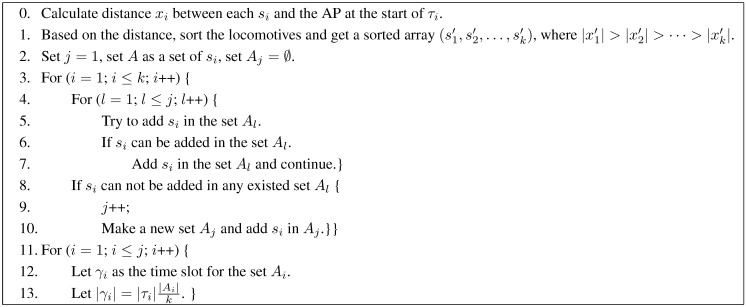
The arranging time slots algorithm for each segment.

To the second step, suppose $s_{i}$ is one of the locomotives in the radius of the AP and there are *k* locomotives during the segment $\tau_{i}$. We will use the following pseudo-code as shown in Figure 4 to calculate each time slot for $s_{i}$.

There are two problems contained in the second step. The first one is how to decide whether $s_{i}$ can be added in the set $A_{l}$? This can be calculated by Equation (4). We have sorted the locomotives based on the distance. The received power on AP can also be sorted based on the distance. That is, the smaller the distance between a locomotive and the AP, the larger the received power on AP would be. When we calculate $s_{i}$ and try to add it in the set $A_{l}$, the power of $s_{j}\left(s_{j}\in A_{l}\right)$ received by AP must be smaller than $s_{i}$’s. Thus, we can calculate the $s_{i}$’s SINR directly by Equation (4), and decide whether it can be added in the set. The second one is how to allocate time slots for each set after we arranged all locomotives in sets for the segment $\tau_{i}$? We believe that if we allocate longer time length for the set with larger number, the received data will then be larger. Thus, we decide it by $|\gamma_{i}\left|=\right|\tau_{i}|\frac{|A_{i}|}{k}$.

In the third step, since the locomotives are traveling in the tunnel, their distances to the AP are always changing, which indicates that their transmitting powers vary over time accordingly. We will calculate them at the start of each segment, first let the locomotive with the maximum distance to the AP transmitting with the maximum power in a set, then the others can be calculated by Equation (4). Suppose $s_{i}$ is the i-th locomotive in a set after sorted, when traveling, the transmitting power can be calculated by Equation (7).

(7) $$\begin{array}{c} P_{i}\left(t\right)=\frac{N_{0}\beta {\left(1+\beta \right)}^{\left(i-1\right)}}{\min\{|v\left(t-t_{s_{i}}\right)-R_{T}{|}^{-\lambda },1\}} \end{array}$$

Now we give a brief analysis to show the time complexity of our algorithm. First of all, the number of time slots is based on the entering and leaving time of each locomotive, so the time complexity would be O(N). Secondly, we should first sort locomotives based on their distances. Many sorting algorithms can be used to do this, and we just suppose we use the bubble sort algorithm with the complexity $O(N^{2})$. Then, we create and arrange the sets $A_{l}$, which needs a double loop, so the complexity is again $O(N^{2})$. Meanwhile, the complexity for calculating $\gamma_{i}$ is O(N). Thirdly, we can calculate the transmitting power scheme for each locomotive directly by using Equation (7), so the time complexity is O(N). Since the first step is used to divide the whole scheduling time into time segments, and in each time segment, we should go through the second and the third steps, therefore, the time complexity for the whole algorithm is $O\left(N\right)\cdot \left(O\left(N^{2}\right)+O\left(N^{2}\right)+O\left(N\right)+O\left(N\right)\right)=O\left(N^{3}\right)$. Notice that in reality the complexity could be much less in that any two locomotives must keep a minimum security distance, which makes the AP can only provide service to certain amount of locomotives within a time slot. Therefore, the number *N* in $O(N^{3})$ may be a much smaller number.

## 4. Simulation Results

In this section, we give simulation results to show the performance of our algorithm. We also compare results with and without SIC to show the advantage of SIC.

We consider wireless networks with 20 to 50 mine locomotives and one AP in a straight tunnel. The position of the AP is zero, and the receiving radius is $R_{T}=120$ m. The transmission powers of the locomotives are between 0 W and 1 W, and the noise power is $N_{0}=10^{-10}$ W. The SINR threshold is β=3. The pass lost index is λ=4. The Channel bandwidth is W=22 MHz. The minimum data transmission rate requirement $r_{i}=500$ Kbps. The traveling speed is v=5 m/s. The distance between two successive locomotives should be kept at least 30 m all the time for safety reasons. Suppose we know all start times $t_{s_{i}}$, which are created randomly in our simulations and satisfy the minimum security distance.

We first present detailed results of a wireless network with 20 locomotives in Section 4.1. Then, we provide complete results for all network instances with different number of locomotives.

### 4.1. Results for a Wireless Network with 20 Locomotives

**Figure 5:**
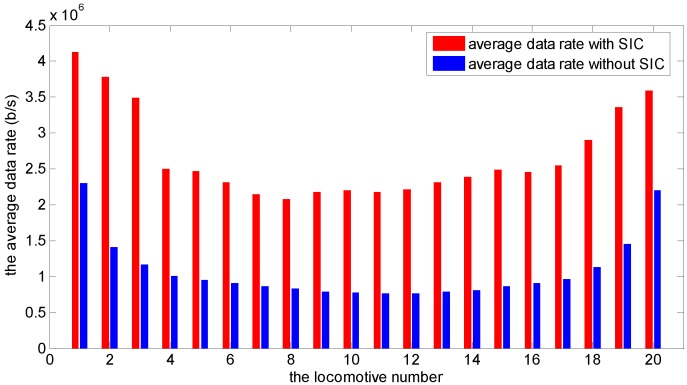
The comparison of average data rate with and without SIC for 20 locomotives.

Consider a wireless network with 20 locomotives. The start times of each locomotive are given in Table 1. Based on the algorithm proposed in Section 3, we can calculate all the locomotive average data rates with and without SIC during the whole scheduling time as shown in Figure 5. From this, we can see, by using SIC, the average data rates are improved significantly. For example, for the first locomotive, the average data rates are 4.13 Mbps by using SIC and 2.30 Mbps when not using SIC. So the scaling factor $K_{1}=8.26$ when using SIC and $K_{1}^{\prime }=4.6$ when not using SIC. The detail data is listed in Table 2. The improvement throughput is $\frac{\sum (K_{i}-K_{i}^{\prime })}{\sum K_{i}^{\prime }}=79.6\%$.

**Table 1:** Start times of the 20 locomotives.

*n*	Start Time (s)	*n*	Start Time (s)	*n*	Start Time (s)	*n*	Start Time (s)
1	0	6	56	11	102	16	146
2	13	7	67	12	108	17	155
3	23	8	76	13	119	18	168
4	37	9	88	14	127	19	181
5	50	10	96	15	138	20	193

**Table 2:** Average data rates and scaling factors with and without successive interference cancellation (SIC) for the 20 locomotives.

*n*	Average Data Rate	Scaling Factor $K_{i}$	Average Data Rate	Scaling Factor $K_{i}^{\prime }$
	with SIC (Mbps)	with SIC	without SIC (Mbps)	without SIC
1	4.13	8.26	2.30	4.60
2	3.78	7.56	1.40	2.80
3	3.49	6.98	1.16	2.32
4	2.50	5.00	1.01	2.02
5	2.46	4.92	0.95	1.90
6	2.31	4.62	0.91	1.82
7	2.15	4.30	0.87	1.74
8	2.07	4.14	0.83	1.66
9	2.17	4.34	0.79	1.58
10	2.20	4.40	0.77	1.54
11	2.18	4.36	0.76	1.52
12	2.21	4.42	0.77	1.54
13	2.32	4.64	0.79	1.58
14	2.39	4.78	0.81	1.62
15	2.49	4.98	0.81	1.62
16	2.46	4.92	0.91	1.82
17	2.55	5.10	0.96	1.92
18	2.90	5.80	1.13	2.26
19	3.36	6.72	1.45	2.90
20	3.60	7.20	2.20	4.40

**Figure 6:**
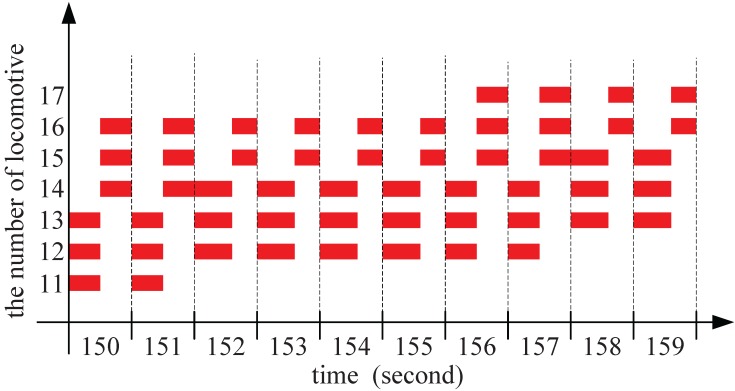
The time slots division from second 150 to second 159.

To show the transmission regularity, we show the time slots division from second 150 to second 159 and draw it in Figure 6. We can see during these ten seconds, seven locomotives are moving along the tunnel. For example, locomotive 11 travels along the tunnel at second 150 and 151, and leaves at second 152. Locomotive 17 enters into the radius of the AP at second 156. In second 155, there are five locomotives moving along the tunnel. Locomotives 12, 13 and 14 are scheduled in a longer time slot, while locomotives 15 and 16, a shorter time slot.

In order to show the time efficiency, we also give some results relating to the time issue here. Since the whole scheduling distance is $2R_{T}=240$ m, while the minimum security distance is 30 m, the number of locomotives that can be it the scheduling time slot at the same time is no greater than 8. The computer used to run our algorithm has an Inter Core i5 CPU with 3.1 GHz and 4 GB memory. We find the whole algorithm can be finished only in several milliseconds for this case, while the whole scheduling time is $t_{s_{20}}^{\prime }-t_{s_{1}}=241$ s.

### 4.2. Results for All Network Instances

**Figure 7:**
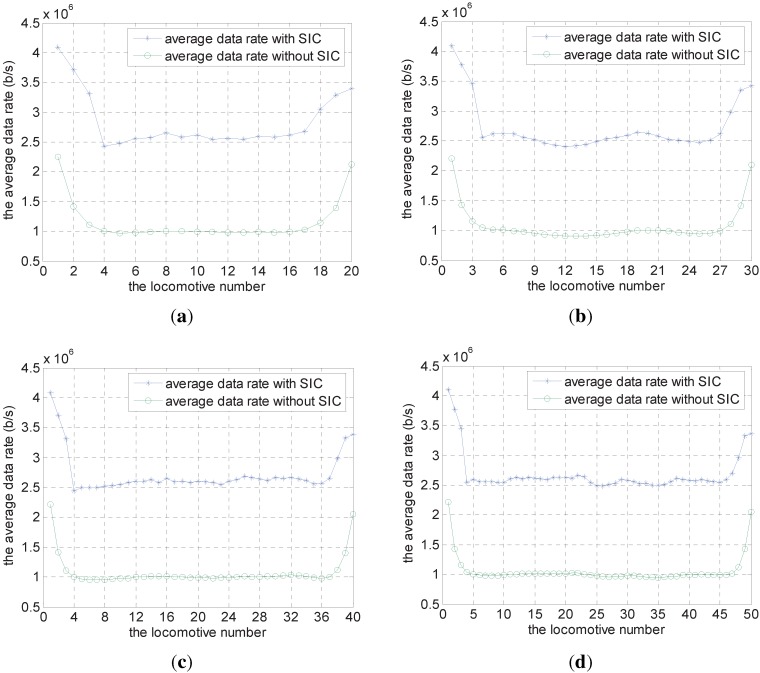
Results for all network instances. (**a**) The number of locomotives is 20; (**b**) The number of locomotives is 30; (**c**) The number of locomotives is 40; (**d**) The number of locomotives is 50.

We change the number of locomotives *n* from 20 to 50, and generate 20 different network instances randomly for each *n*. Then, we calculate the average data rate value for each locomotive under the scheme with and without SIC, and show the results for each *n* in Figure 7. From this, we can see the average data rate for each locomotive under SIC scheme is improved significantly compared with the scheme without SIC. The average improvement throughput is 144.3% with 20 locomotives, 151.6% with 30 locomotives, 152.9% with 40 locomotives and 154.0% with 50 locomotives separately.

## 5. Conclusions

In our previous work, we have applied the SIC technique for interference management and proposed algorithms of link scheduling and data routing schemes for different models [19,20], but these are all based on fixed transmitting nodes. In this paper, we consider that the transmitting nodes keep moving, propose an optimal algorithm and use it in the mine locomotive wireless network successfully. We will further design mine locomotive wireless network strategies with multiple APs and with complicated underground tunnels.
